# An Integrated Approach for the Efficient Extraction and Solubilization of Rice Microsomal Membrane Proteins for High-Throughput Proteomics

**DOI:** 10.3389/fpls.2021.723369

**Published:** 2021-09-09

**Authors:** Truong Van Nguyen, Ravi Gupta, Dicky Annas, Jinmi Yoon, Yu-Jin Kim, Gi Hyun Lee, Jeong Woo Jang, Kang Hyun Park, Randeep Rakwal, Ki-Hong Jung, Cheol Woo Min, Sun Tae Kim

**Affiliations:** ^1^Department of Plant Bioscience, Life and Industry Convergence Research Institute, Pusan National University, Miryang, South Korea; ^2^Department of General Education, College of General Education, Kookmin University, Seoul, South Korea; ^3^Department of Chemistry, Pusan National University, Busan, South Korea; ^4^Department of Life Science & Environmental Biochemistry, Pusan National University, Miryang, South Korea; ^5^Faculty of Health and Sport Sciences, University of Tsukuba, Tsukuba, Japan; ^6^Research Laboratory for Biotechnology and Biochemistry (RLABB), Kathmandu, Nepal; ^7^Graduate School of Biotechnology & Crop Biotech Institute, Kyung Hee University, Yongin, South Korea

**Keywords:** rice, label-free quantitative proteomics, microsomal membrane extraction, ultracentrifugation, AZO, sds, MaxQuant, Perseus

## Abstract

The preparation of microsomal membrane proteins (MPs) is critically important to microsomal proteomics. To date most research studies have utilized an ultracentrifugation-based approach for the isolation and solubilization of plant MPs. However, these approaches are labor-intensive, time-consuming, and unaffordable in certain cases. Furthermore, the use of sodium dodecyl sulfate (SDS) and its removal prior to a mass spectrometry (MS) analysis through multiple washing steps result in the loss of proteins. To address these limitations, this study introduced a simple micro-centrifugation-based MP extraction (MME) method from rice leaves, with the efficacy of this approach being compared with a commercially available plasma membrane extraction kit (PME). Moreover, this study assessed the subsequent solubilization of isolated MPs in an MS-compatible surfactant, namely, 4-hexylphenylazosulfonate (Azo) and SDS using a label-free proteomic approach. The results validated the effectiveness of the MME method, specifically in the enrichment of plasma membrane proteins as compared with the PME method. Furthermore, the findings showed that Azo demonstrated several advantages over SDS in solubilizing the MPs, which was reflected through a label-free quantitative proteome analysis. Altogether, this study provided a relatively simple and rapid workflow for the efficient extraction of MPs with an Azo-integrated MME approach for bottom-up proteomics.

## Introduction

Microsomes are cell membrane-derived vesicles that are formed during the lysis of plant tissues (LaMontagne et al., 2016). They are known to consist of the plasma membrane (PM), endoplasmic reticulum (ER), Golgi apparatus, intracellular vesicles, and tonoplast (Yang and Murphy, 2013; LaMontagne et al., 2016). The successful enrichment and proteomic analyses of microsomes have gained considerable interest in recent years, as researchers aim to understand the functions of numerous membrane proteins that augment our comprehension of the diverse biological pathways operating in different subcellular compartments (Wang et al., 2015; Alqurashi et al., 2017; Meisrimler et al., 2017; Pontiggia et al., 2019). For instance, a recent study investigated various microsomal proteins that were responsive to abscisic acid (ABA) and hydrogen peroxide to understand their mode of action using a shotgun proteomic approach (Alqurashi et al., 2017). However, due to various technical challenges the isolation of microsomal membrane proteins (MPs) remains difficult. This challenge is encountered because these proteins are of low abundance and highly hydrophobic, which is why they are devoid of contaminating molecules such as organic acids, polyphenols, lipids, carbohydrates, and other secondary metabolites that are predominantly present in the plant cells (Cox and Emili, 2006; Vilhena et al., 2015; Mehraj et al., 2018). To date, the large-scale isolation of MPs for proteome analysis has primarily relied on ultracentrifugation-based methods due to multiple advantages such as the higher purity of products, suitability in a large volume of samples, and accurate separation of subcellular compartments based on own sedimentation rates (Yang D. et al., 2020). However, these ultracentrifugation-based methods require expensive instrumentation, skilled technicians, and a large amount of starting material, which limits their large-scale utilization (Wang et al., 2015; Alqurashi et al., 2017; Meisrimler et al., 2017; Yang D. et al., 2020). Thus, commercial kits that facilitate the isolation of MPs and PM proteins have been introduced to address the discussed limitations. However, these kits are often expensive and offer limited extraction, hindering the large-scale preparation of the targeted proteins (Kaundal et al., 2017; Kim et al., 2019; Yang Z. et al., 2020; Kato et al., 2021). Therefore, the development of relatively simpler methods for the isolation of MPs is necessary and is also a prerequisite for microsomal proteomic studies.

There were significant efforts in the past studies that were made to develop approaches for the efficient isolation and identification of MPs without ultracentrifugation (Tu and Kawai, 1998; Cox and Emili, 2006; LaMontagne et al., 2016; Moloney et al., 2016). For instance, the study by Nagahashi et al. (1978) used low-speed centrifugation to separate a crude mitochondrial fraction from the primary roots of barley and showed that about 88% of the mitochondria were sedimented with a centrifugal force of 13,000 *g* (Nagahashi et al., 1978). In a similar study by Giannini et al. (1988), a microsomal membrane fraction was successfully isolated from the roots of red beets (*Beta vulgaris* L. cv. Detroit Dark Red) by centrifugation at 13,000 *g* for 3–4 min, while a further increase in the duration of centrifugation gave a substantial recovery of the tonoplast and PM adenosine triphosphatase (ATPase) (Giannini et al., 1988). However, there has been no research on the implementation of these methods in the isolation of total MPs from plant tissues for large-scale proteomics.

The solubility of extracted MPs is another major concern that hinders their large-scale analysis. Being highly hydrophobic in nature, MPs are currently solubilized either in anionic detergent sodium dodecyl sulfate (SDS), cetyltrimethylammonium bromide (CTAB), 3-[(3-cholamidopropyl)-dimethylammonio]-1-propanesulfonate (CHAPS), or Triton X-100 (Kalipatnapu and Chattopadhyay, 2005). Among these agents, SDS has been a widely used surfactant for the solubilization of MPs in proteomic studies. However, SDS is not compatible with mass spectrometry (MS) as it interferes with chromatographic separation and suppresses peptide ionization during MS analysis (Alfonso-Garrido et al., 2015). Therefore, SDS must be removed prior to MS analysis through rigorous washing, which eventually results in the inevitable loss and degradation of the MPs (Alfonso-Garrido et al., 2015). Fortunately, various MS-compatible surfactants have been developed for the solubilization of proteins. Among these surfactants, 4-hexylphenylazosulfonate (Azo) has shown considerable potential in solubilizing various types of hydrophilic and hydrophobic proteins. Interestingly, Azo can be subjected to rapid degradation through ultraviolet irradiation, and, most importantly, it is compatible with MS analysis (Brown et al., 2019, 2020). The potential of Azo to be used in protein solubilization has already been shown in animal tissues for top-down and bottom-up proteomics studies. Nonetheless, its efficiency in the plant samples still needs to be tested.

In this study, we introduced a microcentrifuge-based method for the enrichment of MPs (MME) using rice as a model system. Furthermore, the extraction efficiency of this method was compared with a commercially available plasma membrane protein extraction (PME) kit using a label-free quantitative proteomic approach. Using the same approach, this study further compared the protein solubilization efficacies of Azo and SDS isolated by the MME method for a bottom-up proteomic analysis.

## Materials and Methods

### Plant Materials and Growth Condition

Rice (*Oryza sativa* L. cv. Dongjin) seeds were sterilized in a 0.05% spotak solution (Bayer Crop Science, Seoul, South Korea) overnight at 28°C and then washed with distilled water five times as described in a previous study (Gupta et al., 2019). The sterilized seeds were then germinated on wet tissue paper at 28°C in the dark and transferred to a Yoshida solution in a growth chamber maintained at 70% humidity at 25°C with a light and dark cycle of 16 and 8 h, respectively (Yoshida et al., 1976). For the proteomic analysis, 4-week-old rice leaves were harvested and immediately stored at –70°C for further analysis.

### Synthesis of the Azo Surfactant

The synthesis of Azo surfactant was carried out as reported in previous studies (Brown et al., 2019, 2020). Briefly, the synthesis involved 0.857 ml of 4 mM of 4-n-hexylaniline (*n* = 4, C6) that was stirred with a mixture of 4.8 ml 10% hydrochloric acid (HCl) and 8 ml of deionized dihydrogen monoxide (H_2_O) at 10°C for 10 min. This was followed by the dropwise addition of 4 ml of 1 mM of ice-cold sodium nitrite (NaNO_2_) into the mixture. After 5 min of stirring at 10°C, 12 ml of 12 mM of sodium carbonate (Na_2_CO_3_) was added dropwise into the solution under constant stirring for an additional 3 min. Afterward, the solution was filtered into a prepared ice-cold flask containing 8 ml of 8 mM of sodium sulfite (Na_2_SO_3_) and incubated at 4°C overnight to allow for the complete precipitation of the surfactant. Finally, the yellow compound was purified through recrystallization, and the structure of Azo was confirmed using electrospray ionization (ESI)-MS and ^1^H-nuclear magnetic resonance (NMR) analyses as described in a previous study (Brown et al., 2019).

### Microsomal Membrane Protein Extraction From Rice Leaves and Western Blot Analysis

The extraction efficiencies of the two different microsomal protein extraction methods, namely, MME and PME (ab65400, Abcam, Cambridge, United Kingdom), were compared. For the MME method, 200 mg of rice leaf powder were homogenized in an High density sucrose (HDS) buffer, which consisted of 37.5 mM of hydroxyethyl piperazine ethane sulfonic acid (HEPES), pH 8, 37.5% (w/w 1.215 M) sucrose, 7.5% (v/v) glycerol, 15 mM of ethylene-diamine-tetraacetic acid (EDTA), 15 mM of ethylene-glycol-tetraacetic acid (EGTA), 1 mM of dithiothreitol (DTT), and a 100 × Halt^TM^ protease inhibitor cocktail (Thermo Fisher Scientific, MA, United States). Afterward, the supernatant was collected after centrifugation at 600 *g* for 3 min at 4°C (Abas and Luschnig, 2010). The obtained pellet was re-extracted, first with half, and then a third of the initially used HDS buffer at the same centrifugation conditions as the first extraction. After centrifugation, both the supernatants were combined and centrifuged again at 600 *g* for 3 min at 4°C to remove any remaining debris. Subsequently, the 1 ml of supernatant was taken out and was diluted with 2.167 ml of double-distilled water (ddH_2_O) to adjust the sucrose to a final concentration of 12–13%. After centrifugation at 21,000 *g* for 90 min at 4°C, the supernatant, referred to as the soluble protein fraction, was precipitated with 12.5% (w/v) trichloroacetic acid (TCA)/acetone containing 0.07% (v/v) β-mercaptoethanol overnight at –20°C and centrifuged at 14,000 *g*. Meanwhile, the pellet, referred to as the microsomal membrane protein fraction, was resuspended in a wash buffer (20 mM HEPES, pH 8.0, 5 mM EDTA, and 5 mM EGTA) and re-centrifuged at 21,000 *g* for 45 min at 4°C. Finally, all of the resulting pellets were additionally washed with 80% acetone and stored –20°C for further analysis. The extraction of MPs using the PME kit was carried out following the instructions of the manufacturer.

A Western blot analysis was performed as described in a previous study (Min et al., 2015). The antibodies, such as the anti-luminal binding protein (BiP), anti-PM intrinsic protein 2-1 (PIP 2-1), and anti-tonoplast intrinsic protein 1-1 (TIP 1-1), were used in the Western blot analysis to confirm the presence of the resident proteins of the Golgi apparatus, PM, and vacuolar membrane, respectively.

### Protein Solubilization and In-Solution Digestion Using the Filter-Aided Sample Preparation Method

The isolated MPs were subjected to in-solution trypsin digestion employing the filter-aided sample preparation (FASP) approach (Wiśniewski et al., 2009; Min et al., 2020a,b). In brief, the acetone-precipitated MPs (300 μg) were dissolved in 30 μl of a denaturation buffer [4% SDS and 100 mM of DTT in 0.1 M of tetraethylammonium tetrahydroborate (TEAB), pH 8.5], sonicated for 3 min, and heated at 99°C for 30 min followed by the loading of the proteins onto a 30K spin filter (Amicon Ultra-0.5 ml, Merck Millipore, Darmstadt, Germany). The protein extract was diluted with a Urea-TEAB (UA) buffer (8 M urea in 0.1 M TEAB, pH 8.5) to a final volume of 300 μl. The protein extract was washed three times with 300 μl of a UA buffer by centrifugation at 14,000 *g* to remove the SDS. Cysteine alkylation was accomplished through the addition of 200 μl of an alkylation buffer [50 mM of iodoacetamide (IAA), 8 M of urea in 0.1 M of TEAB, pH 8.5] for 1 hat room temperature in the dark. This was followed by switching the UA buffer with a TEAB buffer (50 mM of TEAB, pH 8.5) in a spin filter unit. The protein was digested with trypsin [enzyme-to-substrate ratio (w/w) of 1:100] dissolved in 50 mM of the TEAB buffer containing 5% acetonitrile (ACN) at 37°C overnight. The digested peptides were collected by centrifugation, and the filter device was rinsed with 50 mM of TEAB and 50 mM of sodium chloride (NaCl). The procedure for protein solubilization and digestion using the Azo surfactant was similar to the procedures discussed in the previous sections, which used Azo (1% as final concentration) as a replacement for SDS. Prior to reduction and alkylation, the Azo was degraded by exposing the samples to UV-vis irradiation using a 100-W high-pressure mercury lamp (Nikon housing with Nikon HB-10101AF power supply; Nikon, Tokyo, Japan) as described in a previous study (Brown et al., 2019). After the UV irradiation of the samples, the alkylation and incubation with a trypsin solution were applied following the procedures discussed in the previous sections. Finally, the peptide concentrations were measured using the Pierce Quantitative Fluorometric Peptide Assay (Thermo Fisher Scientific, MA, United States) following the instructions of the manufacturer. Further peptide desalting (Gupta et al., 2020; Min et al., 2020b) and pre-fractionation using a basic pH reverse-phase (BPRP) column were also carried out as described in previous studies (Kim et al., 2018; Min et al., 2020b). Detailed procedures for peptide desalting, BPRP peptide fractionation, Q-Exactive Orbitrap liquid chromatography–tandem MS (LC-MS/MS) analysis (Pajarillo et al., 2015; Meng et al., 2019), and data analyses with functional annotations using MaxQuant (Savaryn et al., 2016; Tyanova et al., 2016a), Perseus (Tyanova et al., 2016b), MetaboAnalyst (Chong et al., 2018), AgriGO v2.0 (Tian et al., 2017), CELLO2GO (Yu et al., 2014), and the Kyoto Encyclopedia of Genes and Genomes (KEGG) (Kanehisa et al., 2017; Kanehisa and Sato, 2020) are described in the Supplementary Material. The generated proteomics data were deposited to the ProteomeXchange Consortium *via* the PRIDE partner repository with the dataset identifier PXD025132 (Perez-Riverol et al., 2019).

## Results

### Label-Free Proteomic Analysis of Proteins Isolated by the MME and PME Methods

**FIGURE 1:**
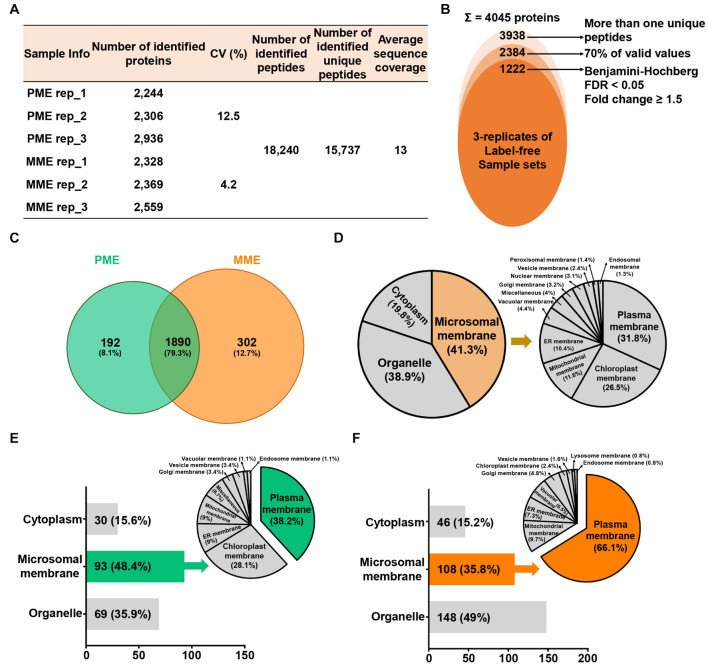
Label-free quantitative proteomic analysis of microsomal membrane proteins (MPs) extracted from rice leaves using the MME and PME methods. **(A)** Table revealing the information of identified proteins in two different sample sets with replicates. **(B)** Circular diagram showing the distribution of the total identified and significantly modulated proteins followed by narrow down approaches. **(C)** Comparison and determination of subcellular localization using 2,384 identified proteins after the removal of potential contaminants. Venn diagram showing the distribution of specifically and commonly identified proteins extracted by the PME and MME methods. Subcellular localization analysis was carried out using commonly **(D)** and uniquely identified proteins in the PME **(E)** and MME **(F)** methods. MME, microsomal membrane protein extraction; PME, plasma membrane extraction kit.

To compare the protein profiles of the isolated MPs, a label-free quantitative proteomic approach was utilized, which led to the identification of 18,240 peptides and 15,737 unique peptides that matched with 4,045 protein groups, resulting in an average sequence coverage of 13% (Figure 1A). Specifically, the three replicates of the same sample in PME showed less than 12.5% of the coefficient of variation (CV) values, while less than 4.2% of the CV values were observed in the case of the MME method (Figure 1A). Further removal of potential contaminants and proteins with >30% missing values (70% valid values in the three replicates in at least one sample) narrowed down the identification list to 2,384 proteins (Figure 1B).

Among these 2,384 proteins, 302 (12.7%) and 192 (8.1%) proteins were uniquely identified in the MME and PME samples, respectively, while 1,890 (79.3%) proteins were commonly identified in both approaches (Figure 1C and Supplementary Table 1). The subcellular localization prediction of commonly identified proteins revealed that 19.8 (375 proteins) and 38.9% (735 proteins) of the proteins were localized in the cytoplasm and organelle, respectively. On the other hand, 41.3% (780 proteins) of the proteins were localized to the cellular membranes as determined with Uniport and the CELLO2GO web-based database (Figure 1D; Yu et al., 2014). The results also showed that, among the 192 proteins specific to the PME method, 30 (15.6%) and 69 (35.9%) proteins were found to be localized in the cytoplasm and organelles, respectively. Furthermore, 93 (48.4%) proteins were determined to be MPs (Figure 1E). Regarding the 302 proteins specific to the MME method, 46 (15.2%) and 148 (49%) proteins were predicted to be localized to the cytoplasm and organelles, respectively. Additionally, 108 (35.8%) proteins were determined to be MPs (Figure 1F). Proteins associated with the PM, chloroplast membrane, ER membrane, mitochondrial membrane, Golgi membrane, vesicle membrane, vacuolar membrane, and endosome membrane were identified in both methods, while lysosome membrane proteins were only identified in the MME method. The PM, chloroplast membrane, ER membrane, and mitochondrial membrane proteins were the major MPs accounting for 34 (38.2%), 25 (28.1%), 8 (9%), and 8 (9%) of the total specific MPs in the PME method, respectively (Figure 1E). Meanwhile, the PM, mitochondrial membrane, ER membrane, and vacuolar membrane proteins were the main MPs, accounting for 82 (66.1%), 12 (9.7%), 9 (7.3%), and 8 (6.5%) of the total specific MPs in the MME method, respectively (Figure 1F). The Western blot analysis of the soluble and microsomal proteins demonstrated a clear and better enrichment of a plasma membrane marker protein, plasma membrane-localized PIP2-1, in the MME as compared with the PME sample (Supplementary Figure 1).

For examining the correlation and variations between the two sample sets and the reproducibility of different replicates of the same sample, multi-scatter plot and principal component (PCA) analyses were performed (Supplementary Figures 2A,B). The multi-scatter plots showed Pearson correlation coefficient values among the same sample ranging from 0.962 to 0.970 and from 0.972 to 0.973 in the three replicates of PME and MME, respectively. This suggests a strong correlation among the replicates of each sample set (Supplementary Figure 2A). In addition, the PCA plot showed a clear separation between the two samples in principal component 1 that accounted for 93.5% of the total variation (Supplementary Figure 2B). The application of a multiple ANOVA test controlled by a Benjamini-Hochberg false discovery rate (FDR) threshold of 0.05 with a fold change of more than 1.5 on the 2,384 high-confidence proteins revealed the identification of 1,222 significantly modulated proteins between the two sample sets. Among these, 614 and 608 proteins showed increased abundance when extracted with the MME and PME methods, respectively (Supplementary Figure 2C).

### Functional Classification of the Identified Proteins

**FIGURE 2:**
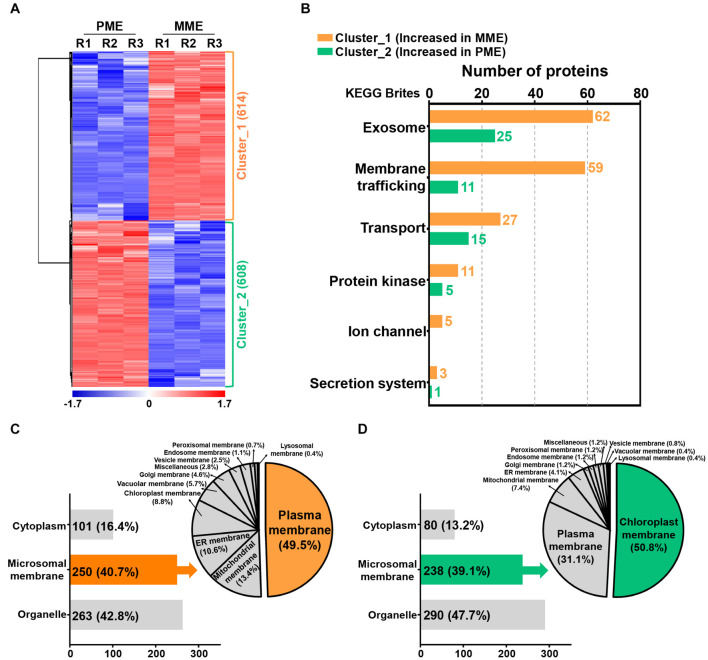
Quantification and functional characterization of the significantly modulated microsomal membrane proteins of rice leaves. **(A)** Hydrophobic cluster analysis (HCA) of 1,222 significantly modulated proteins grouped into two major clusters based on their abundance patterns. **(B)** Functional hierarchical classification of proteins with increased abundance in the MME and PME samples, respectively, using the List of identified Kyoto Encyclopedia of Genes and Genomes (KEGG) brite database. Subcellular localization analysis of significantly modulated proteins is represented as clusters, wherein cluster_1 corresponds to the increased abundance in MME **(C)** and cluster_2 corresponds to the increased abundance in PME **(D)**.

A volcano plot and sequential hydrophobic cluster analysis (HCA) segregated all the significantly modulated proteins into two major clusters. The results revealed that cluster_1, containing 614 proteins, showed an increased abundance in the MME sample and cluster_2, having 608 proteins, had an increased abundance in the PME sample (Figure 2A and Supplementary Table 2). Functional classification of the identified proteins by Gene Ontology (GO) enrichment analysis (Tian et al., 2017) revealed that the intracellular and membrane categories were found to be the most enriched terms in both the clusters in the cellular component category (Supplementary Table 3). In the intracellular category, two subgroups consisting of organelle (with 67 and 86 proteins identified in clusters 1 and 2, respectively) and cytoplasm (comprising 74 and 78 proteins found in clusters 1 and 2, respectively) were enriched in the analysis (Supplementary Table 3). In the membrane category, five different subgroups, namely, intrinsic to the membrane, photosynthetic membrane, organelle membrane, proton-transporting ATPase, and membrane (miscellaneous), were determined in both clusters. However, the associated proteins were majorly enriched in the MME method as compared with the PME method (Supplementary Table 3). Moreover, the membrane coat proteins were specifically identified in the MME sample, while photosynthetic membrane proteins were only identified in the PME sample (Supplementary Table 3).

### Kyoto Encyclopedia of Genes and Genomes and Subcellular Localization Analysis of the Identified Proteins

The Kyoto Encyclopedia of Genes and Genomes (KEGG) pathway analysis using the KEGG brite database (Kanehisa et al., 2017; Kanehisa and Sato, 2020) resulted in the identification of various biological objects including enzymes and other proteins related to the exosome, membrane trafficking, mitochondrial biogenesis, ribosome, and transport, among many others (Supplementary Table 4). In addition, the proteins related to cellular transport reactions including the exosome, membrane trafficking, transport, protein kinase, ion channel, and secretion system showed enriched abundance profiles in the MME sample as compared with the PME sample (Figure 2B and Supplementary Table 5). A total of 62, 59, 27, 11, 5, and 3 proteins showing increased abundances in the MME sample were found to be associated with the exosome, membrane trafficking, transport, protein kinase, ion channel, and secretion system, respectively. These proteins included syntaxin, the SNARE protein, ABC transporter, mitochondrial phosphate carrier protein, sugar transport protein, potassium transporter, mitochondrial outer membrane protein porin, and signal recognition particle receptor subunit alpha, among others. In contrast, only 57 proteins related to cellular transport reactions showed increased abundance in the PME sample (Figure 2B and Supplementary Table 5).

The prediction of the subcellular localization of significantly modulated proteins showed the enhanced abundance of membrane-resident proteins in both the MME and PME samples (Figures 2C,D; Yu et al., 2014). The results showed that, among the 1,222 significantly modulated proteins between the MME and PME methods, 42.8% were localized to various organelles in the samples of the former and 47.7% in those of the latter. However, the MPs were enriched by a similar fraction (39–41%) in both the methods (Figures 2C,D). Microsomal membrane proteins showing an increased abundance in the MME sample were also predicted to be localized in the PM (49.5%), mitochondrial membrane (13.4%), ER membrane (10.6%), chloroplast membrane (8.8%), vacuolar membrane (5.7%), Golgi membrane (4.6%), vesicle membrane (2.5%), endosome membrane (1.1%), peroxisomal membrane (0.7%), lysosomal membrane (0.4%), and other membranes (miscellaneous) (2.8%) (Figure 2C). Meanwhile, the chloroplast membrane-, PM-, mitochondrial membrane-, and ER membrane-associated proteins were the major MPs accounting for 50.8, 31.1, 7.4, and 4.1% of the total specific MPs in the PME method, respectively (Figure 2D). Overall, these results showed that the MME method yielded a highly enriched microsomal protein fraction of rice leaves with minimal chloroplast contamination as compared with the PME method. Moreover, a further comparison using the same approach with the above analysis for the evaluation of the solubilization efficacy of the two different surfactants, Azo and SDS, was carried out. This was carried out to increase the dynamic resolution of the microsomal membrane proteome in the rice leaves.

### Label-Free Quantitative Proteomic Analysis of Azo- and SDS-Solvated Proteins

**FIGURE 3:**
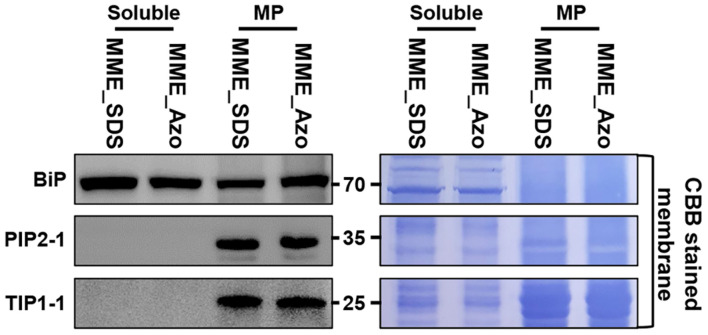
Extraction of microsomal membrane proteins using the MME method and sequentially solubilized in the sodium dodecyl sulfate (SDS) and 4-hexylphenylazosulfonate (Azo) surfactants for label-free quantitative analysis. Further validation of the solubility of microsomal membrane proteins in the SDS and Azo surfactants and Western blot analyses using BiP, PIP2-1, and TIP1-1 antibodies were carried out.

The synthesis and validation of Azo surfactant were carried out following a previous report before conducting the comparative proteomic analysis (Brown et al., 2019). After the affirmation of the mass, the accurate structure (Supplementary Figure 3) and rapid degradation of Azo under UV irradiance (Supplementary Figure 4) and MPs from rice leaves were isolated using the MME method. Subsequently, the obtained MPs were solubilized in either Azo or SDS for comparison. Quantitative analysis showed the solubilization of 96 ± 7 μg of proteins in Azo and 74 ± 9 μg of proteins in the SDS sample per 100 μg of membrane proteins used. This highlighted the better protein solubilization efficiency of Azo compared with SDS. However, subsequent Western blot analyses of the Azo- and SDS-dissolved proteins demonstrated organellar enrichment profiles in the MP fractions utilizing antibodies against membrane-localized BiP, PIP2-1, and TIP1-1 proteins (Figure 3). This suggested the comparable technical efficiencies of SDS and Azo in the solubilization of MPs.

**FIGURE 4:**
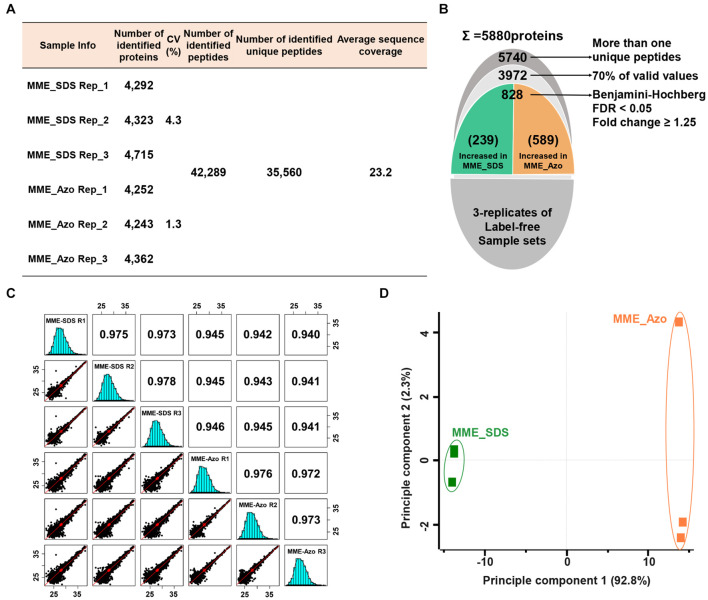
Label-free quantitative proteomic analysis of microsomal membrane proteins solubilized in the SDS and Azo surfactants, respectively. **(A)** Table showing the information of identified proteins in two different sample sets with replicates. **(B)** The narrow-down approach for the identification of significantly modulated proteins. **(C)** Multi-scatter plots represent the Pearson’s correlation coefficient values of each sample solubilized in SDS and Azo. **(D)** Principal component analysis of significantly modulated proteins. MME_SDS, microsomal membrane extraction and dissolved in SDS buffer; MME_Azo, microsomal membrane extraction and dissolved in Azo buffer.

A quantitative proteomic assessment of Azo- and SDS-solubilized MPs (labeled as MME_Azo and MME_SDS, respectively) showed the identification of 42,289 peptides and 35,560 unique peptides matched with 5,880 protein groups (Figures 4A,B). The three replicates of each sample set in MME_SDS and MME_Azo samples revealed CV values less than 4.3 and 1.3%, respectively. This suggested that the MME_Azo sample yielded a much reproducible result as compared with MME_SDS. Furthermore, the sequential downstream processing led to the identification of a total of 3,972 proteins with 70% valid values within three replicates (Figure 4B). Pearson’s correlation coefficient values of the triplicates of the same datasets showed high degrees of correlation and ranged from 0.973 to 0.978 and from 0.972 to 0.976 in the MME_SDS and MME_Azo samples, respectively (Figure 4C). In addition, the PCA plot analysis showed a clear separation between the two samples in principal component 1 that accounted for 92.2% of the total variation (Figure 4D). A multiple ANOVA test controlled by a Benjamini-Hochberg FDR threshold of 0.05 with a fold change of more than 1.25 was applied, which showed a total of 828 significant differentially modulated proteins (Figure 4B and Supplementary Table 6).

### Functional Annotation and Subcellular Localization Analysis of the Identified Proteins

**FIGURE 5:**
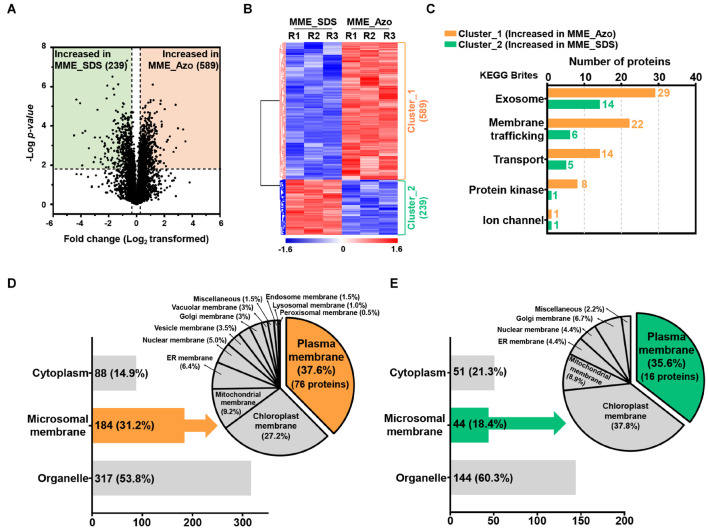
Determination of the abundance profiles and functional classifications of the significantly modulated proteins solubilized in SDS and Azo. Volcano plot **(A)** and HCA **(B)** highlighting the relative fold change differences of the identified proteins from the microsomal membrane fractions solubilized by SDS and Azo. **(C)** The functional classifications of significantly modulated proteins by KEGG pathway analysis using the KEGG brite database. **(D)** Subcellular localization analysis of the proteins with increased abundance in MME_Azo (cluster_1) **(D)** and MME_SDS (cluster_2) **(E)**, respectively. Representatively, three major localizations including the cytoplasm, microsomal membrane, and organelles were predicted by UniProt and the CELLO2GO web-based database.

The volcano plot and HCA of the significantly modulated proteins from the MPs fraction solubilized in two different surfactants were performed to determine protein abundance patterns (Figures 5A,B). The statistically significant proteins were sorted into two major clusters, of which 589 proteins (cluster_1) and 289 proteins (cluster_2) exhibited patterns of increased abundance in MME_Azo and MME_SDS, respectively (Figures 5A,B and Supplementary Table 6). The functional annotation of proteins exhibiting significant differences indicated that intracellular (99 and 67 proteins identified in cluster 1 and 2, respectively) and macromolecular complex (46 and 57 proteins identified in clusters 1 and 2, respectively) were the major enriched GO terms of the cellular component category in both the MME_Azo and SDS samples (Supplementary Table 7). Furthermore, the increased abundance of the 61 proteins related to membrane (GO:0016020) function, including the ABC transporter, transmembrane protein, importin, vesicle transport SNARE protein, mitochondrial carrier protein, and potassium transporter, among others, was uniquely observed in the MME_Azo samples (Supplementary Table 7).

The KEGG analysis further confirmed the functional association of the proteins showing an increased abundance in the MME_Azo samples to the exosome, membrane trafficking, transporter, protein kinase, and ion channel (Figure 5C and Supplementary Tables 8, 9). Subcellular localization showed that, out of 589 proteins showing an increased abundance in MME_Azo (cluster_1), 88 (14.9%) and 317 (53.8%) proteins were predicted to be localized in the cytoplasm and organelles, respectively. On the other hand, 184 (31.2%) proteins were specifically localized to different cellular membranes (Figure 5D). Moreover, 289 proteins showing an increased abundance in the MME_SDS sample (cluster_2) were found to be located at the cytoplasm (51 proteins, 21.3%), organelles (144 proteins, 60.3%), and cellular membranes (44 proteins, 18.4%) (Figure 5E). In particular, the subcellular localization prediction of highly abundant MME_Azo proteins showed their association with the PM (76 proteins, 37.6%), chloroplast membrane (27.2%), and mitochondrial membrane (9.2%). The endoplasmic reticulum, nucleus, vesicle, and Golgi membrane, among others, accounted for 26% of the proteins in the MME_Azo sample (cluster_1) (Figure 5D). In contrast, the highly abundant protein cluster of MME_SDS was distinctly represented by the PM (16 proteins, 35.6%), chloroplast (37.8%), mitochondrial membrane (8.9%), and ER membranes (4.4%), while the remaining organelles were represented by 13.3% of the total proteins in MME_SDS sample (cluster_2) (Figure 5E).

## Discussion

Membranes perform specific functions depending on their associated proteins and carry out the task of demarcating the boundaries between cells and cellular organelles (Luschnig and Vert, 2014; LaMontagne et al., 2016). In particular, microsomal membrane proteins play key roles in essential biological processes such as plant development and tolerance to environmental stressors by activating diverse signaling pathways (Komatsu et al., 2007; Takagi et al., 2011; Gomez-Navarro and Miller, 2016). Some of these signaling events and other functions of MPs are well-characterized, but the majority of these remain understudied because of the technical difficulties in the isolation of these highly hydrophobic proteins. Therefore, this study developed a relatively simpler protocol for the isolation and subsequent solubilization of MPs using rice as a model system. The method is referred to as the MME method in this study. The efficacy of the developed protocol was assessed by two consecutive label-free quantitative proteomic analyses. The foremost analysis dealt with a comparative assessment of the efficacies of the MME and PME methods in the isolation of MPs and the second strategy included a comparison of the solubilization efficiencies using two different surfactants (SDS and Azo surfactants).

The isolation of MPs using ultracentrifugation was first reported by the study of Blackburn and Kasper in 1976, when they used a centrifugation speed of 394,000 *g* for 2 min to isolate insoluble membrane fraction from the rat hepatocytes (Blackburn and Kasper, 1976). In the case of plants, ultracentrifugation was first utilized to purify the PM proteins from barley roots, through which the enrichment of PM proteins was achieved using density gradient centrifugation (Nagahashi et al., 1978). As of today, these ultracentrifugation-based methods are still the most efficient and successful methods for the isolation of MPs from plants (LaMontagne et al., 2016; Meisrimler et al., 2017; Junková et al., 2018). In a recent report, the enrichment of MPs was attempted for the identification of the potential interaction partner of flotillins in *Arabidopsis thaliana* which led to the identification of ATPase 1, early-responsible to dehydration stress protein 4, and syntaxin-71, to be flotillins interacting proteins (Junková et al., 2018). In addition, a study by Meisrimler et al. (2017) employed differential centrifugation for 10 min at 10,000 *g* and 30 min at 50,000 *g* to remove cell debris and soluble proteins, respectively, and subsequently isolate the root-microsomal proteins from four pea cultivars exhibiting variable root lengths during germination (Meisrimler et al., 2017). In particular, the authors identified 33 (55%) MPs associated with the plastid, Golgi membrane, mitochondria, nucleus, tonoplast, and vesicle, of which 20% had a transmembrane region and 27 proteins (45%) were predicted to be localized in the cytoplasm (Meisrimler et al., 2017). Similarly, a few other research groups have also performed the identification and characterization of MPs in plant systems. However, limited efforts have been invested toward the development of a rapid, simple, and reproducible method for plant samples.

**FIGURE 6:**
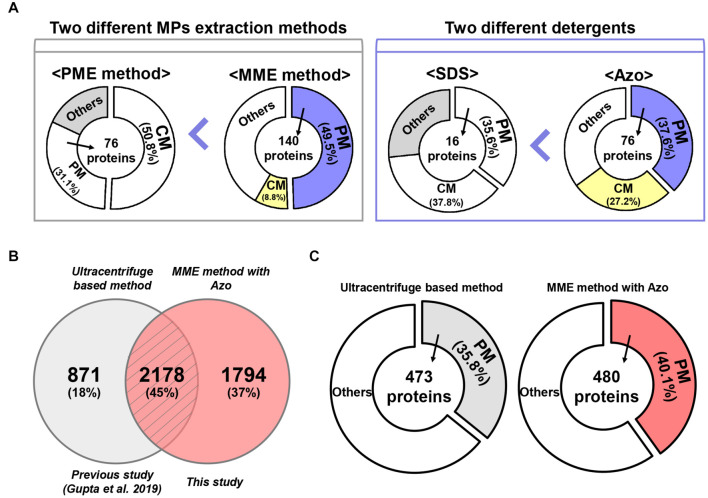
A comparative analysis of MME vs. PME methods and SDS vs. Azo utilizing variable methods for microsomal proteomics. **(A)** The MME method revealed the successful enrichment of MPs, a particularly higher fraction of PM proteins in bottom-up proteomic analysis. Moreover, MPs, especially PM proteins, were solubilized more effectively in Azo and demonstrated differential proteomic identifications when compared with the SDS-based method. **(B)** Venn diagram and **(C)** subcellular localization showing a further comparison of the number of identified proteins in the current study and previous study.

The results of this study suggested that the MME method led to the successful enrichment of several classical MPs as compared with the PME method. Moreover, the majority of the MPs such as sucrose transport protein SUT1, ABC transporter, sugar transporter, receptor-like serine/threonine-protein kinase, calcium transporting ATPase, elicitor responsive protein 1 (ERG1), aquaporin PIP2, sodium/calcium exchanger NCL1, and voltage-gated potassium channel protein, among others, represented higher abundance profiles in the MME methods as compared with the PME method. This difference between the extraction efficiencies of the two methods could be because of the use of different extraction and wash buffers (Figure 6A; Shen et al., 2015; Meisrimler et al., 2017). Furthermore, in this study, we recorded the presence of chloroplast and cytoplasmic proteins in both MME and the PME methods, with the former having a lesser abundance. However, the detection of membrane proteins in most microsomes indicated that chloroplast and cytoplasmic contaminations have minor or no impacts on the identification of MPs. This suggested that the complete removal of chloroplast and cytoplasmic contaminations are somewhat unnecessary. The label-free quantitative proteomic analysis and the functional annotation also provided convincing evidence that the MME method was more effective than the PME method in isolating MPs, leading to an improvement in PM protein recovery.

Plasma membrane proteins contribute a large part of the MPs and play crucial roles in sensing signals, catalysis, transport, adhesion, and construction, which ensure the survival and development of the cells. However, the enrichment of PM proteins is also one of the most significant hurdles for plant proteomics because of their low abundance and high hydrophobicity (Santoni et al., 2000; Kalipatnapu and Chattopadhyay, 2005; Speers and Wu, 2007; Helbig et al., 2010). Conventional methods using ultracentrifugation (centrifugation speed ranging from 40,000 to 156,000 *g*) coupled with sucrose density gradient centrifugation allowed the achievement of relatively pure PM fractions from roots of various plants such as barley, corn, and oats. However, these approaches are known to be complicated and are not adapted universally for green tissues due to unavoidable contaminations by fragmented chloroplasts (Nagahashi et al., 1978; Yoshida et al., 1983; Yang and Murphy, 2013). A previous study focusing on the isolation of transport competent vesicles from plant tissues reported that mitochondrial membrane proteins can be enriched using centrifugation at 13,000 *g* for 3–4 min. In contrast, if the centrifugation was prolonged to 20 min, most of the PM ATPase activity was suspended (Giannini et al., 1988). This procedure nonetheless requires a higher amount of starting material, usually 20–25 g. In the case of this study, only 200 mg or less of starting material was adequate for the isolation of a sufficient amount of MPs for proteome analysis. Moreover, this study also demonstrated that centrifugation time plays a crucial role in the enrichment of microsomal/PM proteins. A higher number of PM proteins were also identified here by the MME method in this study compared with the PME method. This could be due to the prolonged centrifugation (1.5 h) used in the MME method to sediment the PM proteins in our experiment (Giannini et al., 1988).

Protein solubility, especially in the case of MPs, is another major challenge in proteomic studies (Speers and Wu, 2007). Particularly, the anionic detergent SDS has been the most commonly used detergent for the processing of protein samples because it can denature, solubilize, stabilize, and establish proteins separation through SDS-polyacrylamide gel electrophoresis (SDS-PAGE) (Bhuyan, 2010). Although techniques such as exchange liquid chromatography, metal-organic frameworks MIL-101, gel filtration, dialysis, SDS precipitation, affinity spin column, and FASP method have been developed for the effective removal of SDS from protein extracts, SDS at a concentration higher than 0.01% results in a decrease in the signal-to-noise ratio and resolution of the ions of interest during the MS analysis (Wiśniewski et al., 2009; Botelho et al., 2010; Sun et al., 2012; Ilavenil et al., 2016; Žilionis, 2018). To address these problems, we used a photocleavable anionic surfactant, namely, Azo, as a replacement for SDS in the MME method (Brown et al., 2019, 2020). Our results showed that the solubilization of MPs in Azo was advantageous over SDS as the number, amount, and abundance of MPs were higher in the MME_Azo sample as compared with the MME_SDS sample. Interestingly, 184 proteins in the MME_Azo sample were found to be MPs, with 76 proteins, including ABC transporter, aquaporin, sugar transporter protein MST4, transmembrane receptor family protein, potassium transporter, and voltage-gated potassium channel protein, among others, were specifically localized to the PM (Figure 6A). In the case of SDS solubilized proteins, only 16 PM proteins were identified out of 44 MPs (Figure 6A). A comparison of the identified MPs was made with a previous study that characterized various MPs associated with plant-pathogen interaction from rice leaves using an ultracentrifugation-based method (Gupta et al., 2019). Results showed 45% of the commonly identified MPs in both studies of which 18% (871 proteins; Gupta et al., 2019) and 37% (1,794 proteins; this study) of the MPs were specifically identified in each study, respectively (Figure 6B). Besides, a subcellular localization analysis of the total identified proteins in each study revealed that 480 PM proteins (40.1%) were identified specifically in the current study (Figure 6C), while only 473 (35.8%) of the proteins were found to be located at PM in the previous study (Figure 6C; Gupta et al., 2019). Further subcellular localization analysis of the identified proteins between the two studies provided convincing evidence that the MME method may comparatively be useful for isolating various MPs, especially for the enrichment of the PM proteins. Moreover, the results from the present study have suggested that Azo can effectively solubilize the MPs from rice leaves for bottom-up proteomics with a similar or higher performance that is comparable with that of SDS. Further, the advantageous effects of Azo in solubilizing various membrane-localized proteins have been validated with respect to SDS, which is in concordance with a similar methodology followed in the study by Brown et al. (2019) in animals. Since Azo is compatible with MS analysis and can easily be degraded under UV radiation, it can be safely implemented as an SDS replacement for bottom-up proteomics (Brown et al., 2020).

In conclusion, this study reported a simple, reproducible, and cost-effective method for the isolation of MPs for bottom-up proteomics. So far, Azo has only been used in the proteomics field for the top-down proteomics of animal proteins. Therefore, this is the first study in which the efficacy of Azo was shown in the solubilization of plant proteins for a bottom-up proteomic analysis (Brown et al., 2019). The Azo-integrated MME approach has its advantages, including being simple, time-saving, and easy to scale up in laboratory conditions. Furthermore, it is also highly effective in isolating and solubilizing the MPs with performance comparable with that of the ultracentrifugation-based methods.

## Data Availability Statement

The original contributions presented in the study are publicly available. This data can be found here: PRIDE (ID: PXD025132).

## Author Contributions

RG, CWM, and STK conceptualized the experiments. TVN, GHL, and JWJ prepared samples for the proteomic analysis. DA and KHP synthesized the Azo surfactant with ESI-MS and ^1^H-NMR analyses. CWM, JY, and Y-JK performed the functional analysis of the proteomic data. TVN, CWM, RR, RG, and K-HJ wrote and made the English corrections. All authors read and agreed to the published version of the manuscript.

## Conflict of Interest

The authors declare that the research was conducted in the absence of any commercial or financial relationships that could be construed as a potential conflict of interest.

## Publisher’s Note

All claims expressed in this article are solely those of the authors and do not necessarily represent those of their affiliated organizations, or those of the publisher, the editors and the reviewers. Any product that may be evaluated in this article, or claim that may be made by its manufacturer, is not guaranteed or endorsed by the publisher.

## Supplementary Material

Supplementary Figure 1 Validation of the extraction efficiencies of microsomal membrane proteins using the PME and MME methods, respectively, and the Western blot analysis using the plasma membrane-localized PIP2-1 antibody was carried out.

Supplementary Figure 2 Statistical validation of the label-free quantitative proteomic analysis. **(A)** Pearson correlation coefficient values of the triplicates of each sample by a multi-scatter analysis. **(B)** Principal component analysis of significantly modulated proteins. **(C)** Volcano plot highlighting the relative fold change between the samples.

Supplementary Figure 3 Structural validation of the synthesized Azo surfactant. The mass **(A)** and structure **(B,C)** of the Azo surfactant were measured by electrospray ionization–tandem MS (ESI-MS/MS) and ^1^H-nuclear magnetic resonance (^1^H-NMR) analyses.

Supplementary Figure 4 Photo-degradation kinetics of the Azo surfactant upon UV irradiation with normal **(A)** and heating **(B)** conditions after dissolving in a denaturation buffer.

Supplementary Table 1 Identification of 2,384 proteins by label-free quantitative analysis using microsomal proteins extracted by two different methods in rice leaves.

Supplementary Table 2 Identification of differentially modulated proteins by comparison between two different microsomal protein extraction methods.

Supplementary Table 3 Gene ontology (GO) enrichment analysis of differentially modulated proteins by comparison between two different microsomal protein extraction methods.

Supplementary Table 4 List of identified KEGG brite using differentially modulated proteins by comparison between two different microsomal protein extraction methods.

Supplementary Table 5 KEGG pathway analysis of differentially modulated proteins by comparison between two different microsomal protein extraction methods using the KEGG brite database.

Supplementary Table 6 Identification of differentially modulated proteins by comparison between the samples dissolved in different surfactants.

Supplementary Table 7 GO enrichment analysis of differentially modulated proteins by comparison between the samples dissolved in two different surfactants.

Supplementary Table 8 List of identified KEGG brite using differentially modulated proteins by comparison between the samples dissolved in two different surfactants.

Supplementary Table 9 KEGG pathway analysis of differentially proteins by comparison between the samples dissolved in two different surfactants using the KEGG brite database.
