# Supplementary material for: An Integrated Approach for the Efficient Extraction and Solubilization of Rice Microsomal Membrane Proteins for High-Throughput Proteomics
Source: Front Plant Sci. 2021 Sep 9;12:723369. doi: 10.3389/fpls.2021.723369 (PMC8460067; doi:10.3389/fpls.2021.723369)
Supplement: Supplementary Figure 1 — Validation of the extraction efficiencies of microsomal membrane proteins using the PME and MME methods, respectively, and the Western blot analysis using the plasma membrane-localized PIP2-1 antibody was carried out. [file Data_Sheet_1.PDF]

Supplementary Materials

1 Supplementary Methods

1.1 Peptide desalting, BPRP peptide fractionation, and Q-Exactive MS analysis

The peptide samples were desalted using the HLB OASIS column as described in previous reports and following the manufacturer's instructions (Gupta et al., 2020; Min et al., 2020). After desalting, the dried peptides were reconstituted in 200 µL of loading solution (15 mM Ammonium formate, 2% ACN) and loaded onto stage-tip prepared by packing C18 Empore disk membranes (3M, Bracknell, UK) at the bottom and POROS 20 R2 reversed phase resin into 200 µL yellow tip. Before loading the peptides, the stage-tip was washed with 100% methanol, 100% ACN and equilibrated with loading solution. The peptides were loaded, and 3 fractions subsequently eluted with pH 10 buffer solution series containing 5, 10, 15, 20, 25, 30, 35, 40, 60, 80, and 100% ACN (Min et al., 2020). Finally, the 3 fractions were lyophilized in a vacuum centrifuge and stored at -80°C for further LC-MS/MS analysis as described previously (Kim et al., 2018). Briefly, the obtained peptides were re-dissolved in 2% ACN and 0.1% formic acid (solvent-A) and separated by reversed-phase chromatography using a UHPLC Dionex UltiMate ® 3000 (Thermo Fisher Scientific, MA, USA) instrument (Pajarillo et al., 2015; Gupta et al., 2019). For trapping the sample, the UHPLC was equipped with Acclaim PepMap 100 trap column (100 µm × 2 cm, nanoViper C18, 5 µm, 100 Å) and subsequently washed with 98% solvent A for 6 min at a flow rate of 6 µL/min. The sample was continuously separated on an Acclaim PepMap 100 capillary column (75 µm × 15 cm, nanoViper C18, 3 µm, 100 Å) at a flow rate of 400 nL/min. The LC analytical gradient was run at 2% to 35% solvent-B (100% ACN and 0.1% formic acid) for 90 min, 35% to 95% for 10 minutes, followed by 90% solvent-B for 5 minutes, and finally 5% solvent B for 15 minutes. Liquid chromatography-tandem mass spectrometry (LC-MS/MS) was coupled with an electrospray ionization source to the quadrupole-based mass spectrometer QExactive™ Orbitrap High-Resolution Mass Spectrometer (Thermo Fisher Scientific, MA, Waltham, USA) with a Top15 method with the same parameters as described in previous reports (Min et al., 2019). Thereafter, the proteomics data was deposited to the ProteomeXchange Consortium via the PRIDE partner repository with the dataset identifier PXD025132 (Perez-Riverol et al., 2019).

1.2 Data analysis by MaxQuant and Perseus software

The obtained MS data were analyzed using MaxQuant software (version 1.6.17.0) as described previously (Savaryn et al., 2016; Tyanova et al., 2016a). All three technical replicates of MS/MS spectra were cross-referenced against the *Oryza sativa* database (Oryza sativa pepV6, 67,393 entries). Label-free quantification (LFQ) data were processed using default precursor mass tolerances set by Andromeda, with 20 ppm for the first search and 4.5 ppm for the following ones. The LFQ data was searched based on 0.5 Da of a product mass tolerance with a maximum of two missed tryptic digestions. Carbamidomethylation of cysteine residues was selected for the fixed modifications, while acetylation of lysine residues and oxidation of methionine residues were chosen for additional modifications. The false discovery rate (FDR), set at 1% for peptide identifications, was determined based on a reverse nonsense version of the original database.

Statistical analysis and quantile normalization of LFQ data were carried out using Perseus (ver. 1.6.14.0) and MetaboAnalyst (Ver. 4.0) software, respectively (Tyanova et al., 2016b; Chong et al., 2018). The Perseus software allows data analysis using several parameters including missing values imputation of protein intensities from a normal distribution (width: 0.3, downshift: 1.8), hierarchical clustering analysis (HCA), and multiple sample test (one-way ANOVA) controlled by Benjamini-Hochberg FDR threshold of 0.05, and determination of significant differences in the protein abundance (≥ 1.5-fold change) amongst different samples. The functional classification, Gene Ontology (GO) enrichment, subcellular localization, and KEGG pathway analysis were performed using AgriGO v2.0, CELLO2GO, and KEGG brite web-based database, respectively (Yu et al., 2014; Kanehisa et al., 2017; Tian et al., 2017; Kanehisa and Sato, 2020).

2 References

Chong, J., Soufan, O., Li, C., Caraus, I., Li, S., Bourque, G., et al. (2018). MetaboAnalyst 4.0: Towards more transparent and integrative metabolomics analysis. *Nucleic Acids Res.* 46, W486–W494. doi:10.1093/nar/gky310.

Gupta, R., Min, C. W., Kim, S. W., Yoo, J. S., Moon, A. R., Shin, A. Y., et al. (2020). A TMT-based quantitative proteome analysis to elucidate the TSWV induced signaling cascade in susceptible and resistant cultivars of *Solanum lycopersicum*. *Plants* 9:290. doi:10.3390/plants9030290.

Gupta, R., Min, C. W., Kim, Y. J., and Kim, S. T. (2019). Identification of Msp1-induced signaling components in rice leaves by integrated proteomic and phosphoproteomic analysis. *Int. J. Mol. Sci.* 20:4135. doi:10.3390/ijms20174135.

Kanehisa, M., Furumichi, M., Tanabe, M., Sato, Y., and Morishima, K. (2017). KEGG: New perspectives on genomes, pathways, diseases and drugs. *Nucleic Acids Res.* 45, D353–D361. doi:10.1093/nar/gkw1092.

Kanehisa, M., and Sato, Y. (2020). KEGG Mapper for inferring cellular functions from protein sequences. *Protein Sci.* 29, 28–35. doi:10.1002/pro.3711.

Kim, D. K., Park, J., Han, D., Yang, J., Kim, A., Woo, J., et al. (2018). Molecular and functional signatures in a novel Alzheimer’s disease mouse model assessed by quantitative proteomics. *Mol. Neurodegener.* 13:2. doi:10.1186/s13024-017-0234-4.

Min, C. W., Jung, W. Y., Park, H. J., Moon, K. B., Ko, H., Sohn, J. H., et al. (2019). Label-free quantitative proteomic analysis determines changes in amino acid and carbohydrate metabolism in three cultivars of *Jerusalem artichoke* tubers. *Plant Biotechnol. Rep.* 13, 111–122. doi:10.1007/s11816-019-00518-3.

Min, C. W., Park, J., Bae, J. W., Agrawal, G. K., Rakwal, R., Kim, Y., et al. (2020). In-depth investigation of low-abundance proteins in matured and filling stages seeds of *Glycine max* employing a combination of protamine sulfate precipitation and TMT-based quantitative proteomic analysis. *Cells* 9:1517. doi:10.3390/cells9061517.

Pajarillo, E. A. B., Kim, S. H., Lee, J. Y., Valeriano, V. D. V., and Kang, D. K. (2015). Quantitative proteogenomics and the reconstruction of the metabolic pathway in *Lactobacillus mucosae* LM1. *Korean J. Food Sci. Anim. Resour.* 35, 692–702. doi:10.5851/kosfa.2015.35.5.692.

Perez-Riverol, Y., Csordas, A., Bai, J., Bernal-Llinares, M., Hewapathirana, S., Kundu, D. J., et al. (2019). The PRIDE database and related tools and resources in 2019: Improving support for quantification data. *Nucleic Acids Res.* 47, D442–D450. doi:10.1093/nar/gky1106.

Savaryn, J. P., Toby, T. K., and Kelleher, N. L. (2016). A researcher's guide to mass spectrometry-based proteomics. *Proteomics* 16, 2435–2443. doi:10.1002/pmic.201600113.

Tian, T., Liu, Y., Yan, H., You, Q., Yi, X., Du, Z., et al. (2017). AgriGO v2.0: A GO analysis toolkit for the agricultural community, 2017 update. *Nucleic Acids Res.* 45, W122–W129. doi:10.1093/nar/gkx382.

Tyanova, S., Temu, T., and Cox, J. (2016a). The MaxQuant computational platform for mass spectrometry-based shotgun proteomics. *Nat. Protoc.* 11, 2301–2319. doi:10.1038/nprot.2016.136.

Tyanova, S., Temu, T., Sinitcyn, P., Carlson, A., Hein, M. Y., Geiger, T., et al. (2016b). The Perseus computational platform for comprehensive analysis of (prote)omics data. *Nat. Methods* 13, 731–740. doi:10.1038/nmeth.3901.

Yu, C. S., Cheng, C. W., Su, W. C., Chang, K. C., Huang, S. W., Hwang, J. K., et al. (2014). CELLO2GO: A web server for protein subCELlular lOcalization prediction with functional gene ontology annotation. *PLoS One* 9:e99368. doi:10.1371/journal.pone.0099368.

3 Supplementary Figures

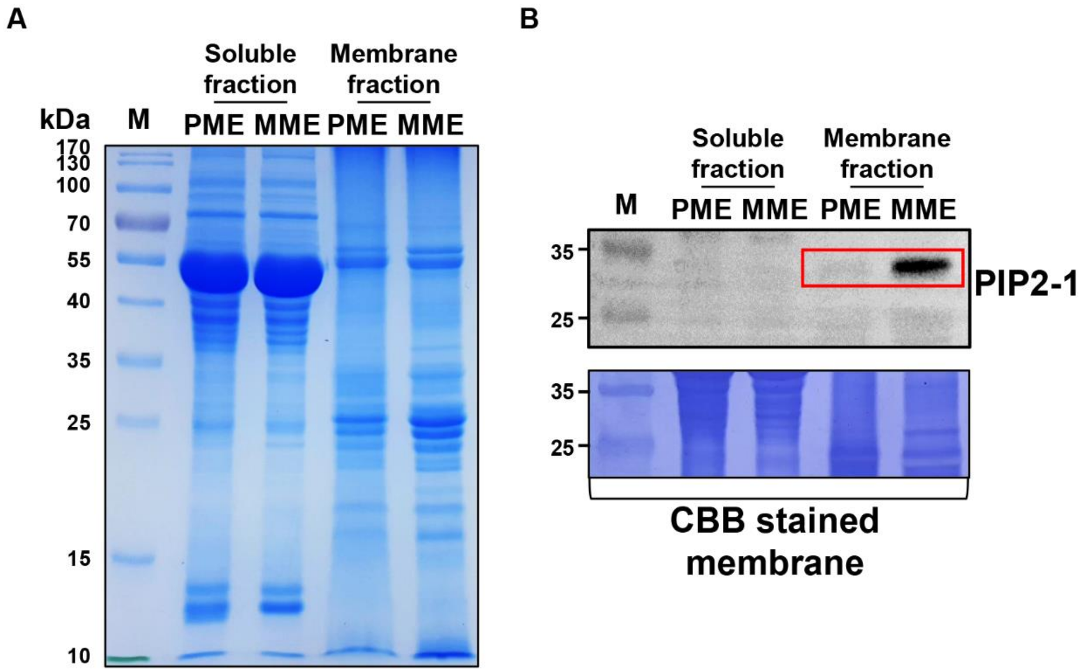

Figure S1. Validation of extraction efficiency of microsomal membrane proteins using PME and MME methods, respectively, the Western blot analysis using plasma membrane-localized PIP2-1 antibody was carried out.

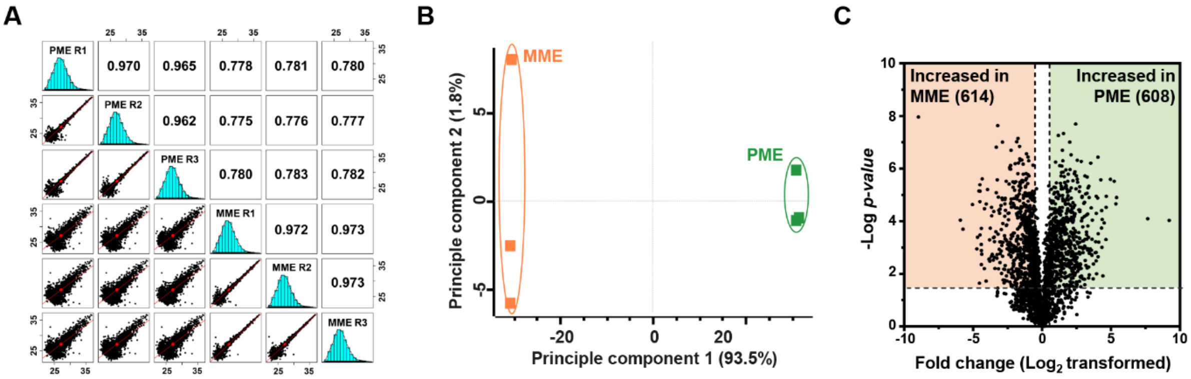

Figure S2. Statistical validation of label-free quantitative proteomic analysis. (A) Pearson correlation coefficient values of triplicates of each sample by multi-scatter analysis. (B) Principle component analysis of significantly modulated proteins. (C) Volcano plot highlighting the relative fold change between the samples.

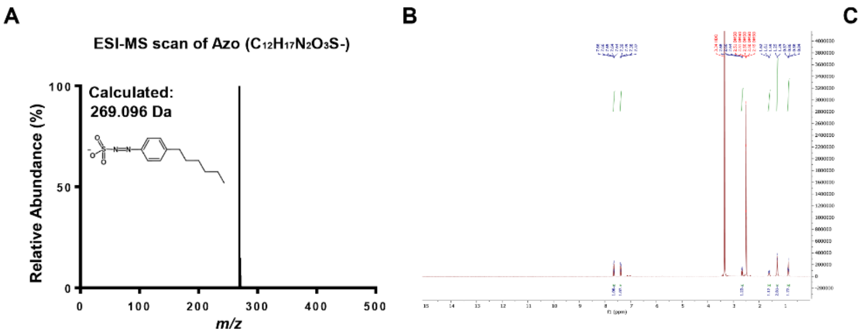

C

Shift (ppm)	Multiplicity	
7.66	dd	2H, -Ar-H
7.39	d	2H, -Ar-H,
2.68	m	2H, -Ar-CH2
1.62	t	2H, t, -Ar-CH2CH2
1.29	t	6H, t, -(CH2)3
0.87	t	3H, -(CH2)3-CH3

Figure S3. Confirmation of structural and mass of Azo surfactant. The mass (A) and structure (B and C) of the Azo surfactant were measured by ESI-MS/MS and ^{1}H-NMR analyses.

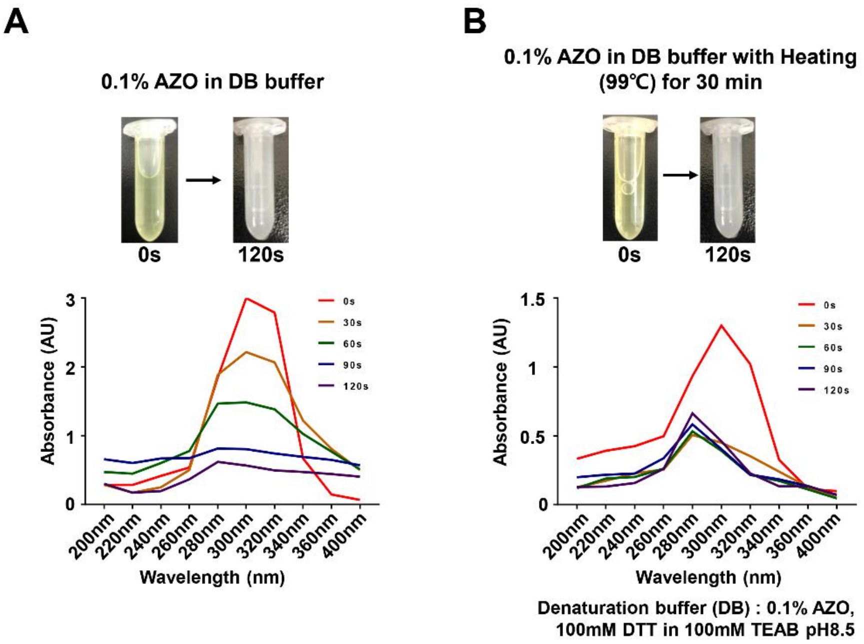

Figure S4. Photo-degradation kinetics of Azo surfactant upon UV irradiation with normal (A) and heating (B) condition after dissolving in denaturation buffer.
